# Dual-pathway mechanism of vanadium-induced hepatotoxicity in ducks: Synergistic crosstalk between glucose homeostasis disruption and NADH/FSP1/COQ10 axis-driven ferroptosis

**DOI:** 10.7150/ijbs.123482

**Published:** 2026-01-01

**Authors:** Huawei Chen, Xueyan Dai, Zhiwei Xiong, Huabin Cao, Chenghong Xing, Haotang Li, Xiaona Gao, Mingwen Hu, Fan Yang

**Affiliations:** Jiangxi Provincial Key Laboratory for Animal Health, Institute of Animal Population Health, College of Animal Science and Technology, Jiangxi Agricultural University, No. 1101 Zhimin Avenue, Economic and Technological Development District, Nanchang 330045, Jiangxi, PR China.

**Keywords:** vanadium, glucose homeostasis, NADH/FSP1/CoQ_10_ axis, ferroptosis, MAMs proteomics

## Abstract

In intensive duck production systems, vanadium (V) is widely used as a growth-promoting additive, but excessive supplementation poses health risks to ducks. Previous research indicated that V could cause damage to organs by disrupting the structure and function of mitochondria and the endoplasmic reticulum. However, the precise mechanism of mitochondrial-associated endoplasmic reticulum membranes (MAMs) in V-induced hepatotoxicity remains unclear. To fill this gap, this study employed network toxicology to analyze the hepatotoxicity of V, and further validated the pivotal roles of glucose homeostasis and ferroptosis in this process through targeted MAMs proteomics. The results indicated that V exposure increased liver dysfunction markers, disrupted hepatic cord structure, and widened ER-mitochondria gaps. Besides, V exposure up-regulated the levels of the IP3R-Grp75-VDAC1 complex in MAMs while promoting its dissociation. Moreover, the sequencing results of MAMs demonstrated that V primarily induced hepatotoxicity by disturbing the glycolysis/gluconeogenesis pathway. Notably, V exposure exacerbated lipid peroxides and Fe^2+^ accumulation while inhibiting the NADH/FSP1/CoQ_10_ axis, down-regulating the expression levels of ferroptosis-related factors in livers. These findings demonstrated that dietary V overexposure impaired hepatic MAMs integrity, disrupted glucose homeostasis, and suppressed the NADH/FSP1/CoQ_10_ axis, which ultimately induced ferroptosis-mediated liver injury in ducks.

## 1. Introduction

Vanadium (V), a widely existing chemical element in the natural environment, is utilized in steel production, energy storage, titanium alloy manufacturing, and numerous other fields. Its critical role in modern industry and national defense has earned it the nickname “metal vitamin” 1. Recently, the burgeoning demand for new energy batteries has accelerated the exploitation and utilization of V resources. However, this intensive utilization has led to the discharge of substantial quantities of V waste into the environment, ultimately resulting in severe contamination of both aquatic and terrestrial ecosystems. Research has revealed that approximately 10% of groundwater samples collected from California and several other states in the United States exhibited V concentrations surpassing 25 μg/L, far exceeding the allowable limit of 10 μg/L for V in drinking water 2, 3. The concentration of V in the soil of regions in China affected by V mining and smelting has soared to an astonishing 4800 mg/kg, far exceeding the average abundance (135 mg/kg) of V in soil 4, 5. Under the context of intensive poultry farming, the toxic effects of vanadium pollutants accumulating in the environment on poultry health have attracted increasing concern 6, 7. Our previous research demonstrated that exposure to V disrupted the biological processes regulated by multiple organelles and activated a cascade of programmed cell death mechanisms, including autophagy and apoptosis, which ultimately resulted in hepatic damage 8, 9. In recent years, the study of cellular organelle interactions has emerged as a research hotspot in life sciences, serving as a key entry point for exploring heavy metal toxicology mechanisms. However, the precise role of organelle interactions in V-induced hepatotoxicity remains elusive to date. Therefore, it is imperative to conduct an in-depth investigation of V-induced hepatotoxicity from the perspective of organelle interactions, thereby unveiling the intricate organ toxicity associated with V.

Mitochondrial-associated endoplasmic reticulum membranes (MAMs) serve as a bridge for information exchange between mitochondria and the endoplasmic reticulum (ER), representing a critical focus in the study of organelle interactions. Our previous research has unveiled that V disrupted the integrity of MAMs, thereby activating ER quality control mechanisms and ultimately triggering cellular apoptosis and autophagy 10-12. Despite these advancements, the intricate mechanisms underlying MAMs-mediated V-induced liver injury remain largely unexplored and enigmatic. Therefore, performing proteomic sequencing on subcellular organelle membranes, particularly those of the MAMs, is of paramount importance for gaining profound insights into the intricate mechanisms of intracellular signal transduction and the intricate architecture of regulatory networks. In this study, we conducted a quantitative proteomic analysis of MAMs extracted from duck livers and found a close association between V exposure and MAM-mediated cellular metabolism.

As is well known, the structural integrity of MAMs is fundamental to cellular metabolic processes. It has been reported that heavy metals exposure could lead to decreased membrane fluidity, increased membrane permeability, and loss of protein function in MAMs through various mechanisms, such as interactions with membrane lipids and binding to membrane proteins 13. Alterations in the structure and function of MAMs severely disrupt the inter-organellar material transport processes, impairing their regulatory capacity over metabolic enzyme activity and subcellular localization, ultimately leading to the imbalance of multiple energy substrate (lipids, glucose, and proteins) homeostasis 14. Among these substrates, glucose homeostasis serves as the fundamental mechanism underlying the stability of intracellular energy supply and is closely linked to the toxic effects of various environmental pollutants, including heavy metals, pesticides, and chemical raw materials. Furthermore, the research indicates that glucose homeostasis is crucial for the stability of organismal functions, and its imbalance can lead to a series of programmed cell deaths 15. Therefore, we hypothesized that heavy metals may disrupt the structure of MAMs, leading to glucose homeostasis imbalance and further inducing cell death. Heavy metal exposure can trigger many types of cell death, and the imbalance of glucose homeostasis during this process is increasingly becoming a central concern in contemporary scientific research. Existing research has demonstrated that V possessed physiological functions similar to insulin, exhibiting potential for regulating glucose homeostasis 16. Notably, disruption of glucose homeostasis can lead to the abnormal accumulation of a series of metabolic substances, subsequently inducing excessive lipid peroxidation and triggering ferroptosis. Yu Zhang et al. 17 confirmed that V oxides could induce glucose homeostasis imbalance through dual inhibitory pathways involving glycolysis and the pentose phosphate pathway, leading to glutathione (GSH) depletion and the induction of ferroptosis. Nonetheless, the precise function of glucose homeostasis in this process remains unclear. Hence, the underlying mechanisms of ferroptosis, particularly its link to glucose homeostasis in V-exposed duck livers, require further exploration.

Currently, with the extensive exploitation and utilization of V, the environmental pollution and biological hazards associated with V cannot be overlooked. In this study, ducks were employed as model animals to establish a V-exposure model. We extracted MAMs from duck livers and conducted quantitative proteomic analysis, validating the metabolic pathways enriched in this crucial lipid raft domain. Our findings revealed the potential molecular mechanisms underlying MAM-mediated V-induced liver injury, thereby offering a novel perspective in the field of environmental toxicology for investigating the toxicity of V exposure in duck livers.

## 2. Materials and Methods

### 2.1 Animals and treatments

A total of 108 Peking ducks (1-day-old, half-male and half-female) were randomly allocated into three groups (36 ducks per group) and were housed according to protocols approved by Jiangxi Agricultural University Animal Care and Use Committee (Approval ID: JXAULL-2024-09-02). The ducks were housed in individual, ventilated cages under specific pathogen-free, environmentally controlled conditions (temperature range: 20-25°C; humidity: 65%), with unrestricted access to both water and feed. The ducks were randomly assigned into four groups: the control group (basal diet), the 30 mg/kg V group (basal diet + 30 mg/kg V) the 45 mg/kg V group (basal diet + 45 mg/kg V) a 7-day adaptation period prior. The NH_4_VO_3_ (99% purity, Analytical reagent grade, supplied by XiLong Scientific, China) served as the supplementary form of V. The dosage of V was determined based on the LD_50_ obtained from a prior study 18. On the 42^nd^ day post-challenge, serum and liver samples were collected from ducks.

### 2.2 Network toxicology

A network toxicology approach was employed to investigate the potential mechanisms of vanadium-induced liver injury. First, genes associated with “vanadium” and “liver injury” were retrieved from the Comparative Toxicogenomics Database (CTD; https://ctdbase.org/) and the GeneCards database (https://www.genecards.org/), respectively. Subsequently, the gene sets from both sources were cross-compared to identify potential candidate genes involved in vanadium-induced liver injury. Finally, Gene Ontology (GO) and Kyoto Encyclopedia of Genes and Genomes (KEGG) pathway enrichment analyses were performed on the overlapping genes using the Genedenovo platform (https://www.omicshare.com/tools/).

### 2.3 Sample collection and MAMs fractionation

On day 42 of the experiment, liver tissue samples were collected from ducks after deep anesthesia by an overdose of intravenous sodium pentobarbital. Following dissection, a portion of the liver tissue was aliquoted into cryovials, flash-frozen in liquid nitrogen, and subsequently transferred to a -80°C ultra-low temperature freezer for long-term storage. The pretreated liver tissue samples were then stored in 4% formaldehyde solution (stored at room temperature), and 2.5% glutaraldehyde solution (stored at 4℃) for subsequent analysis. MAMs fraction was isolated from duck hepatocytes with the modified method using a Percoll gradient 10, 19. Detailed steps for extracting the MAMs were provided in Supplementary Information 1.

### 2.4 Morphological examination

The procedures adhered to the methodology outlined in the previous study 20, 21. After 24 h of fixation in 4% paraformaldehyde, duck liver tissue sections were dehydrated, transparent, embedded, and sectioned. Hematoxylin and eosin staining (H&E) and periodic acid-schiff staining (PAS) were used for histopathological analysis. The case viewer software was utilized to observe the staining outcomes. Additionally, the ultrastructural features of duck livers were examined through transmission electron microscopy (TEM) 22, 23.

### 2.5 DAB-enhanced Perls' Prussian blue staining

Hepatic iron level was measured by DAB-enhanced Perls' Prussian blue stain kit (Leagene, DJ0010). According to the manufacturer's instructions, liver tissue sections are dewaxed in xylene and then soaked in perls stain for 15-30 min. Then stain with DAB stain for 5-10 min and soak with DAB buffer. Then stained with hematoxylin for 1-2 min, conventional dehydration was carried out, and the tablets were sealed with gum. The fluorescence microscope (Olympus DX51, Japan) was used to capture the images, which were then analyzed using Image-Pro Plus software.

### 2.6 Biochemical parameters detection

The contents of aspartate transaminase (AST), alanine transaminase (ALT), γ-glutamyl transferase (GGT), total bilirubin (TBIL), and glucose (GLU) of duck serum were measured with an automated chemistry analyzer (HITACHI 7600, Japan).

### 2.7 Immunofluorescence analysis

Immunofluorescent detection was performed using antibodies against inositol 1,4,5-trisphosphate (IP3) receptor (IP3R) (ABclonal, 1:500, China), glucose-regulated protein 75 (Grp75) (Upingbio, 1:1000, China), voltage-dependent anion channel 1 (VDAC1) (ABclonal, 1:1000, China). The tissue sections were first incubated with their respective primary antibodies. After washing off excess antibody with phosphate-buffered saline, they were incubated with a secondary antibody, specifically goat anti-rabbit or anti-mouse IgG (Proteintech, 1:10000, China). Following another PBS wash, the sections were stained with 4',6-diamidino-2-phenylindole (Beyotime, China). Finally, the fluorescence intensities were visualized using a Nikon Eclipse C1 fluorescence microscope (Tokyo, Japan).

### 2.8 Immunohistochemistry analysis

Refer to previous research methods for specific steps 24. The sections were dewaxed and dehydrated for antigen repair. Then, adding the autofluorescence quencher after 5 min, they were incubated with 5% bovine serum albumin for 30 min. Then, the primary antibody against hydroxynonenal (4-HNE) (Servicebio, 1:500, China), Ferroptosis suppressor protein 1 (FSP1) (Proteintech, 1:500, China), and Glutathione peroxidase 4 (GPX4) (Servicebio, 1:1000, China) was incubated at 4°C for 24 hours. After incubation with horseradish peroxidase (HRP) labeled secondary antibody for 50 min, incubated with 3, 3-diaminobenzidine solution. Section images were obtained by optical microscope (Olympus, Japan).

### 2.9 The oxidative stress index, total iron and ferrous iron (Fe^2+^) levels determination

Glutathione (GSH) and malonaldehyde (MDA) contents were determined strictly on the basis of the kit's instructions (Nanjing Jiancheng Bioengineering Institute, China) 25. After the liver was weighed, a representative portion of tissue was transferred into a 1.5 mL EP tube, to which normal saline or an appropriate buffer was added in a defined mass-to-volume ratio. Homogenization was performed using a homogenizer at 4°C (1500 r/min), repeated 4-5 times, with each cycle lasting 5-10 S and intervals of 1 min between cycles. Total iron and Fe^2+^ levels were measured by the colorimetric assay kit (Elabscience, Wuhan). Briefly, fresh liver tissues were homogenized in reagent I and centrifuged at 10000 × g for 10 min, the supernatant was collected into 1.5 ml Eppendorf (EP) tubes. Then reagent II was added to the EP tube, mixed well and incubated at 4℃ for 40 min. Subsequently, after centrifugation at 12000 × g for 10 min, the supernatant was taken into the wells of the microplate, and the total iron and Fe^2+^ levels were determined by fluorescence at 593 nm using a microplate reader.

### 2.10 CoQ_10_ and CoQ_10_H_2_ contents measurement

CoQ_10_ and CoQ_10_H_2_ contents were measured by the ELISA kit (Meimian, Jiangsu). Briefly, fresh liver tissues were homogenized in saline and centrifuged at 3000 × g for 10 min. The supernatant sample was dispensed into a microplate and allowed to incubate with the enzyme conjugate for 60 min at a temperature of 37℃. After five washes with the specified washing solution, substrates A and B were introduced and incubated for another 15 min at 37℃. Subsequently, a stop solution was administered to each well, and the levels of CoQ_10_ and CoQ_10_H_2_ were determined by measuring the fluorescence emission at 450 nm using a microplate reader.

### 2.11 NADH and NAD+ contents measurement

NADH and NAD+ contents were measured by the colorimetric assay kit (Elabscience, wuhan). Briefly, fresh liver tissues were homogenized with reagent I, centrifuged at 12000 × g for 10 min, and the supernatant was filtered through a 10 KDA ultrafiltration tube. The filtered liquid was added to the assay wells corresponding to the microplate, and the reaction working solution was added and incubated in a 37℃ incubator for 10 min. At the end of the incubation, reagent three was added to the wells and incubated again for 10 min. At the end of incubation, NADH and NAD+ levels were measured by fluorescence at 450 nm using a microplate reader.

### 2.12 Tandem mass tags (TMT) labeling quantitative proteomic analysis

Proteins were extracted and analyzed quantitatively in accordance with the previous study 10. The TMT labeling-based quantitative proteomics workflow employed in this study consisted of the following key steps: Proteins extracted from samples were first digested into peptides, which were then labeled with multiplexed TMT reagents. After labeling, peptides from different samples were pooled in equal amounts. To reduce sample complexity, the mixed peptides were fractionated using high-pH reverse-phase chromatography. The fractions were subsequently analyzed by liquid chromatography-tandem mass spectrometry (LC-MS/MS). Protein identification was achieved by searching the mass spectrometry data against a protein database using specialized software, and accurate quantification across samples was performed based on the intensity of TMT reporter ions. TMT labeling quantitative proteomic analysis for MAMs fraction extracted from liver tissues was executed by Gene Denovo Technology Co., LTD (Guangzhou, China). TMT proteomics analysis was performed with 3 technical replicates.

### 2.13 Real-time quantitative PCR (RT-qPCR)

The total RNA was isolated from liver tissues employing TriZol reagent, subsequently synthesized into cDNA through the use of a reverse transcription kit 26. The RT-qPCR analysis was carried out according to the previously described protocol 27, 28. The primers for β-actin, IP3R, MFN2, Grp75, VDAC1, PACS2, AKR1A1, ALDH1A3, PGK1, ENO1, ENO2, TPIS, G6pase, FBP1, PCK1, PCK2, PTGS2, FSP1 and GPX4 are listed in Table 1.

### 2.14 Western blotting analysis

The protocol of the western blotting assay was in line with the method previously 29, 30. Grp75 (ABclonal, 1:5000, China), IP3R (ABclonal, 1:5000, China), VDAC1 (ABclonal, 1:2500, China), AKR1A1 (Upingbio, 1:2500, China), ALDH1A3 (Upingbio, 1:2500, China), TPIS (Upingbio, 1:2500, China), ENO1 (Upingbio, 1:2500, China), PCK1 (Upingbio, 1:2500, China), G6pase (Upingbio, 1:2500, China), FBP1 (Upingbio, 1:2500, China), PTGS2 (Abmart,1:2500, China), FSP1 (Proteintech, 1:2500, China), GPX4 (Abmart,1:2500, China), and β-actin (Abmart, 1:5000, China) antibodies were employed.

### 2.15. Protein-protein interaction analysis

To analyze the protein-protein interactions (PPIs) of the differentially expressed proteins identified in our study, we utilized the STRING database, accessible at https://cn.string-db.org/.

### 2.16 Statistical analysis

Quantitative variables are presented as mean ± standard deviation (SD). Statistical analyses were conducted using one-way ANOVA and the LSD test through SPSS 25.0 software (SPSS Inc., USA) and GraphPad Prism 8.0 (GraphPad Inc., USA). Significance was set at *P* < 0.05.

## 3. Results

### 3.1 Network toxicology analysis of the potential mechanisms of V-induced liver damage

**Figure 1:**
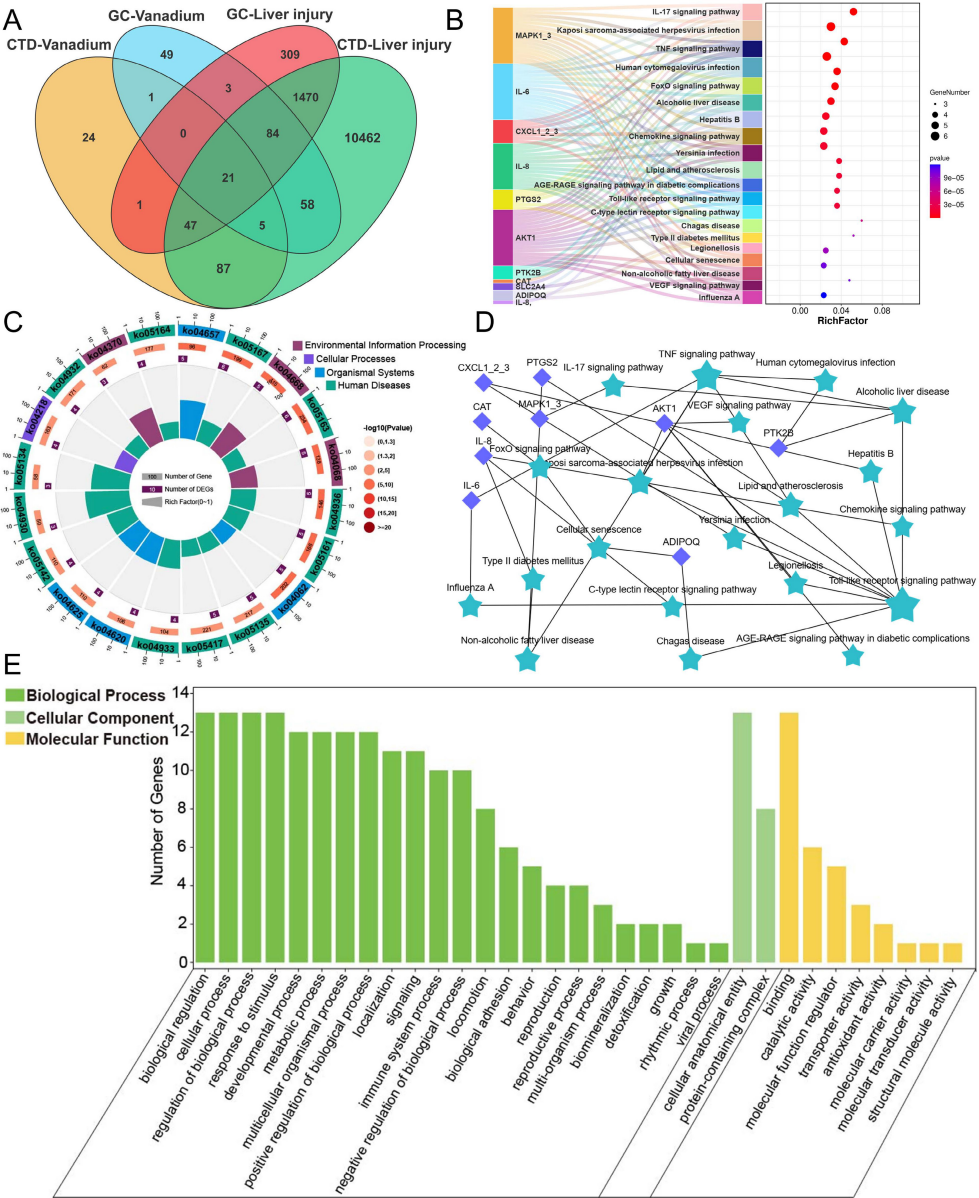
V network toxicology analysis results. (A) Venn diagram for screening V-induced liver damage related genes. (B) KEGG enrichment results of V-induced liver damage related genes. (C) KEGG enrichment circle diagram of V-induced liver damage related genes. (D) PPI network diagram of V-induced liver damage related genes. (E) Level 2 GO terms of V-induced liver damage related genes.

Utilizing the CTD and GeneCards databases and identifying intersections, we screened out 27 V-related target genes and 1,622 liver injury-related target genes. Further intersection of these datasets revealed 21 overlapping target genes (Figure 1A). Further kyoto encyclopedia of genes and genomes (KEGG) pathway analysis of the target genes revealed that they were primarily enriched in the IL-17 signaling pathway, TNF signaling pathway, FoxO signaling pathway, Alcoholic liver disease pathway, Hepatitis B pathway, and Chemokine signaling pathway (Figure 1B-D). Gene Ontology (GO) enrichment analysis showed that, in terms of biological process (BP), the genes were primarily associated with biological regulation, cellular processes, response to stimulus, and metabolic processes. For cellular component (CC), the genes were localized in cellular anatomical entities and protein-containing complexes. Regarding molecular function (MF), the genes were enriched in categories such as binding, catalytic activity, and molecular function regulator (Figure 1E). These results indicated that V primarily influenced biological processes such as hepatocyte biological regulation, stimulus response, and cellular metabolism by regulating pathways related to hepatocyte apoptosis, alcoholic liver disease, and chemokines, thereby inducing liver injury.

### 3.2 V exposure induced liver injury in ducks

**Figure 2:**
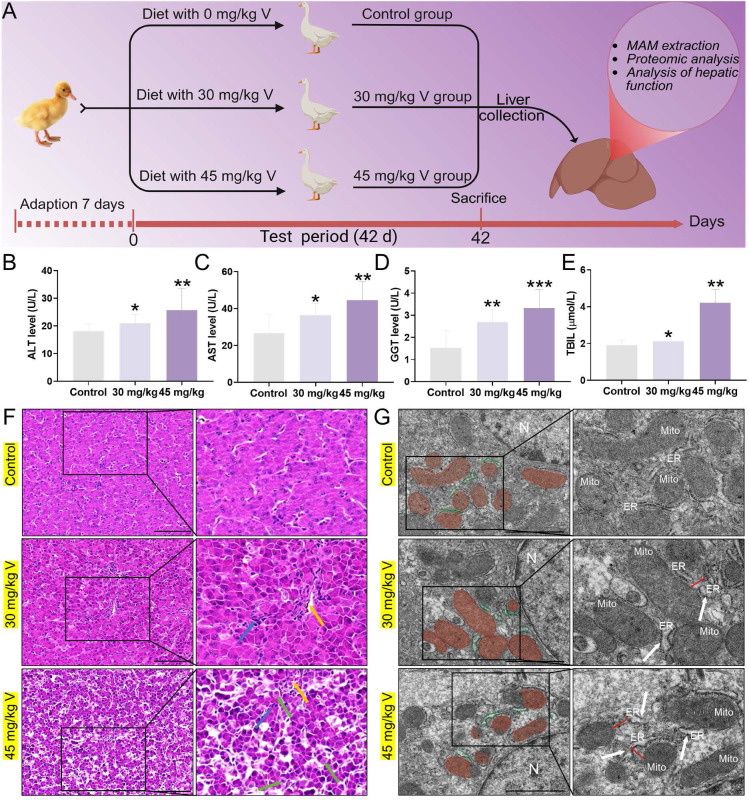
V exposure induced liver injury in ducks. (A) Schematic of the experimental strategy. (B) ALT level. (C) AST level. (D) GGT level. (E) TBIL level. (F) Histopathological observation. The yellow arrows indicated hepatic sinusoids hemorrhage, the bule arrows indicated inflammatory cells infiltration in the livers and the green arrows indicated hepatocyte vacuolation, Scale bar: 100 μm. (G) Ultrastructure observation. The white arrows indicated the swelling of the endoplasmic reticulum, and the red line segments indicated an increase in the distance between the ER and the mitochondria, N: nucleus, Mito: mitochondria, ER: endoplasmic reticulum, Scale bar: 1 μm.

The schematic diagram of the V treatment model in this experiment was as shown in Figure 2A. Firstly, we measured key serum biochemical indicators (ALT, AST, GGT, TBIL) to assess hepatic function. The results showed that V treatment significantly increased the levels of these indicators compared with the control group (*P* < 0.05, *P* < 0.01, or *P* < 0.001), with the most prominent effect observed in the high-dose group (Figure 2B-E). The results of HE staining indicated that the hepatocytes were arranged regularly, with normal morphology and a visible hepatic cord structure in the control group. In contrast, as the therapeutic dose of V gradually increased, the liver tissues exhibited a series of pathological changes: the hepatic cords became disordered; the hepatic sinusoids showed mild dilation with a small amount of red blood cells retained within them (yellow arrows). Meanwhile, within the lobular areas, we also observed infiltration of inflammatory cells (blue arrows). Notably, in the 45mg/kg V group, there was an additional occurrence of mild edematous degeneration in hepatocytes, which was manifested as loose cytoplasm with pale staining (green arrows) (Figure 2F). Transmission electron microscopy revealed that the ER exhibited swelling (white arrows), and the interstitial space between its membrane and the outer mitochondrial membrane had increased after V treatment (red line segments). At the same time, mitochondria cristae had become blurred and damaged (Figure 2G). The aforementioned results demonstrated a dose-dependent induction of liver injury by V in ducks.

### 3.3 V exposure induced the dissociation of the IP3R/Grp75/VDAC1 complex in duck live

**Figure 3:**
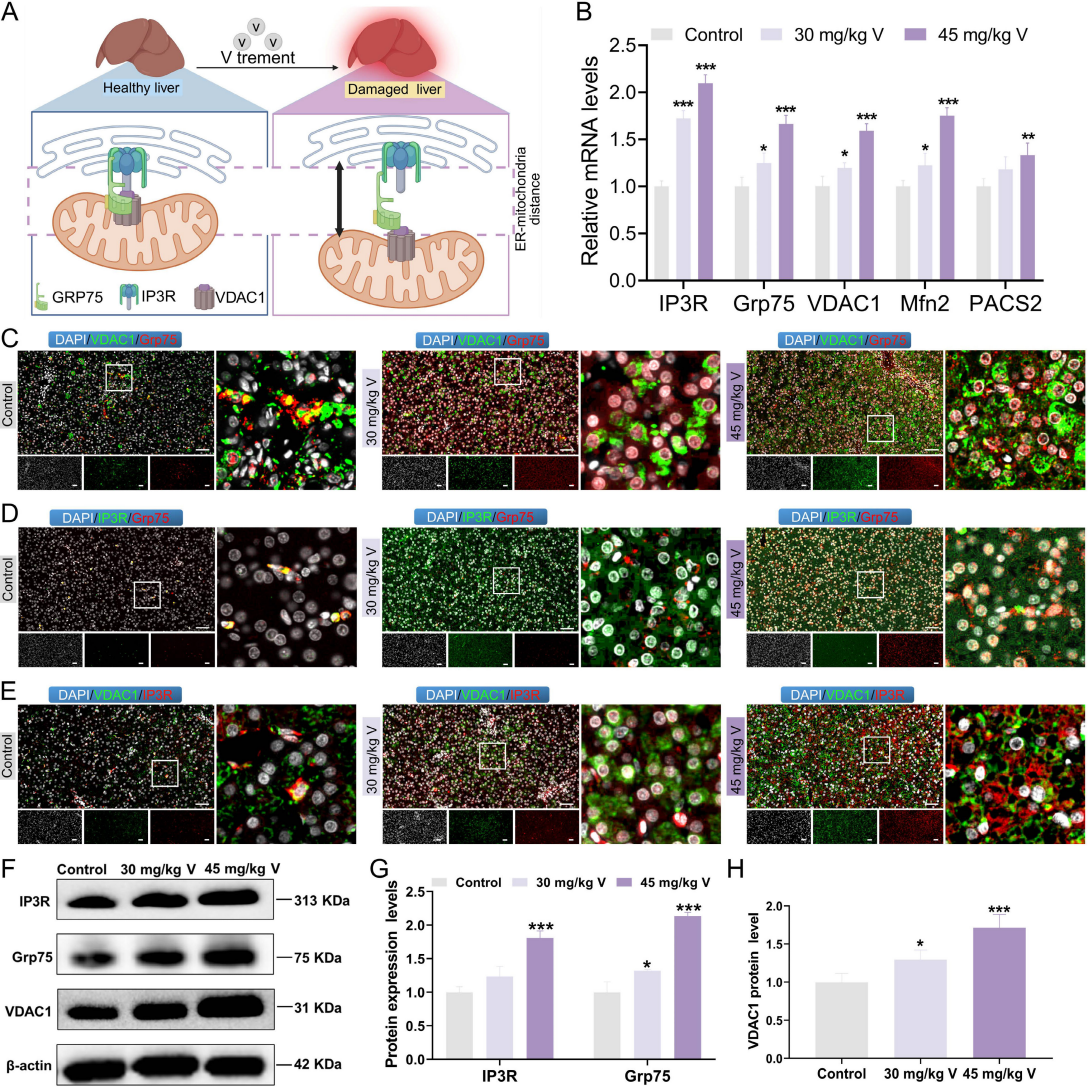
The interconnectivity in the IP3R/Grp75/VDAC1 complex was enhanced under V exposure in duck livers. (A) Schematic diagram of dissociation of the IP3R/Grp75/VDAC1 complex. (B) The relative mRNA levels of IP3R, Grp75, VDAC1, Mfn2, and PACS2. (C)The immunofluorescence colocalization images between VDAC1 and Grp75, scale bar is 50 μm. (D) The immunofluorescence colocalization images between IP3R and Grp75, scale bar is 50 μm. (E) The immunofluorescence colocalization images between VDAC1 and IP3R, scale bar is 50 μm. (F) The images of MAMs associated protein levels (IP3R, Grp75, VDAC1 and β-actin). (G, H) Gray value analysis. “*” indicated *P* < 0.05, “**” indicated *P* < 0.01 and “***” indicated *P* < 0.001 vs. Control group. The same scheme also applies to the remaining figures.

The IP3R/Grp75/VDAC1 complex plays a crucial role in maintaining the structure and function of MAMs (Figure 3A). To explore the changes in IP3R/Grp75/VDAC1 complex, the immunofluorescence co-localization analysis was used in duck livers. The results indicated that the fluorescence intensities of Grp75, VDAC, and IP3R in the 30 mg/kg and 45 mg/kg V-treated groups were significantly increased, compared with the control group. However, the density of yellow fluorescent spots indicating colocalization among these three proteins decreased under V exposure (Figure 3C-E). Subsequently, the MAMs-related mRNA and protein levels were detected. As illustrated in Figure 3B, and Figure 3F-H, compared with the control group, the mRNA levels of IP3R, Grp75, VDAC1, and Mfn2 notably increased in both 30 mg/kg and 45 mg/kg V-treated groups, whereas PACS2 mRNA was only significantly up-regulated in the 45 mg/kg V group (*P* < 0.05, *P* < 0.01 or *P* < 0.001) (Figure 3B). Moreover, the protein expression levels of IP3R, Grp75, and VDAC1 exhibited a conspicuous growth in both the 30 mg/kg and 45 mg/kg V-treated groups (*P* < 0.05, *P* < 0.01 or *P* < 0.001) (Figure 3F-H). These results indicated that V exposure increased the expression levels of the IP3R/Grp75/VDAC1 complex and enhanced the dissociation of its components.

### 3.4 Protein identification and function classification of MAMs from V-treated liver of ducks

**Figure 4:**
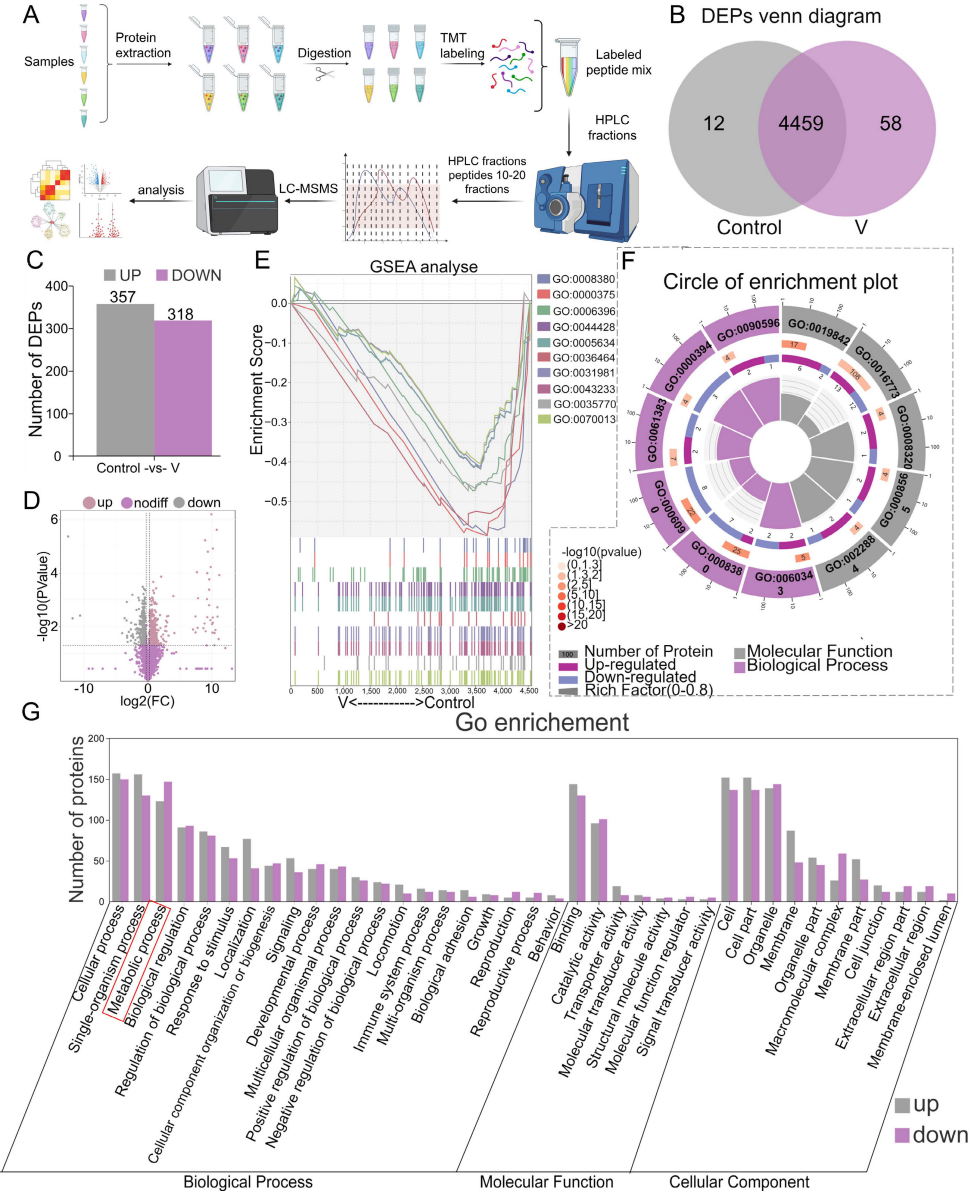
Protein identification and GO annotation of MAMs in duck livers. (A) Flow chart of quantitative proteomic analysis approach utilizing TMT labeling. (B) DEPs venn diagram. (C) Bar chart of DEPs. (D) Volcano Plot for differential protein expression in MAMs. (E) GSEA analysis plot of the top 10 GO-enriched pathways. (F) Circle of enrichment plot of the top 11 GO-enriched pathways. (G) Categorization of differentially expressed proteins based on their functional roles.

The MAMs proteins extracted from duck livers were isolated and subsequently identified through proteomic analysis utilizing TMT (Figure 4A). A total of 4529 proteins were identified during this analysis (Figure 4B). Among these, 357 differentially accumulated proteins (DEPs) were found to be up-regulated, while 318 were down-regulated, as illustrated by the bar chart plot and volcano Plot (Figure 4C-D). Further classification of these MAMs-associated DEPs in both control and V-exposed groups was conducted using GO databases, categorizing them into Biological Process (BP), Cellular Component (CC), and Molecular Function (MF) (Figure 4G). BP analysis revealed that V-exposure induced changes in cellular processes, single-organism processes, metabolic processes, and biological regulations, among others. The MF analysis indicated that these DEPs were primarily involved in binding, catalytic activity, molecular transducer and transporter activity, and so forth. CC analysis demonstrated that the V-induced DEPs were localized to cell parts, cells, organelles, membranes, and other cellular structures. Additionally, the results of Gene Set Enrichment Analysis (GSEA) revealed that the top 10 GO-enriched pathways were activated in the V-exposed group (Figure 4E). From a functional perspective, 6 out of 11 pathways belonged to BP categories, while 5 belonged to MF categories, highlighting the diverse functional impacts of V-exposure on the MAMs proteome (Figure 4F).

### 3.5 V exposure disturbed MAMs proteostasis in duck livers

**Figure 5:**
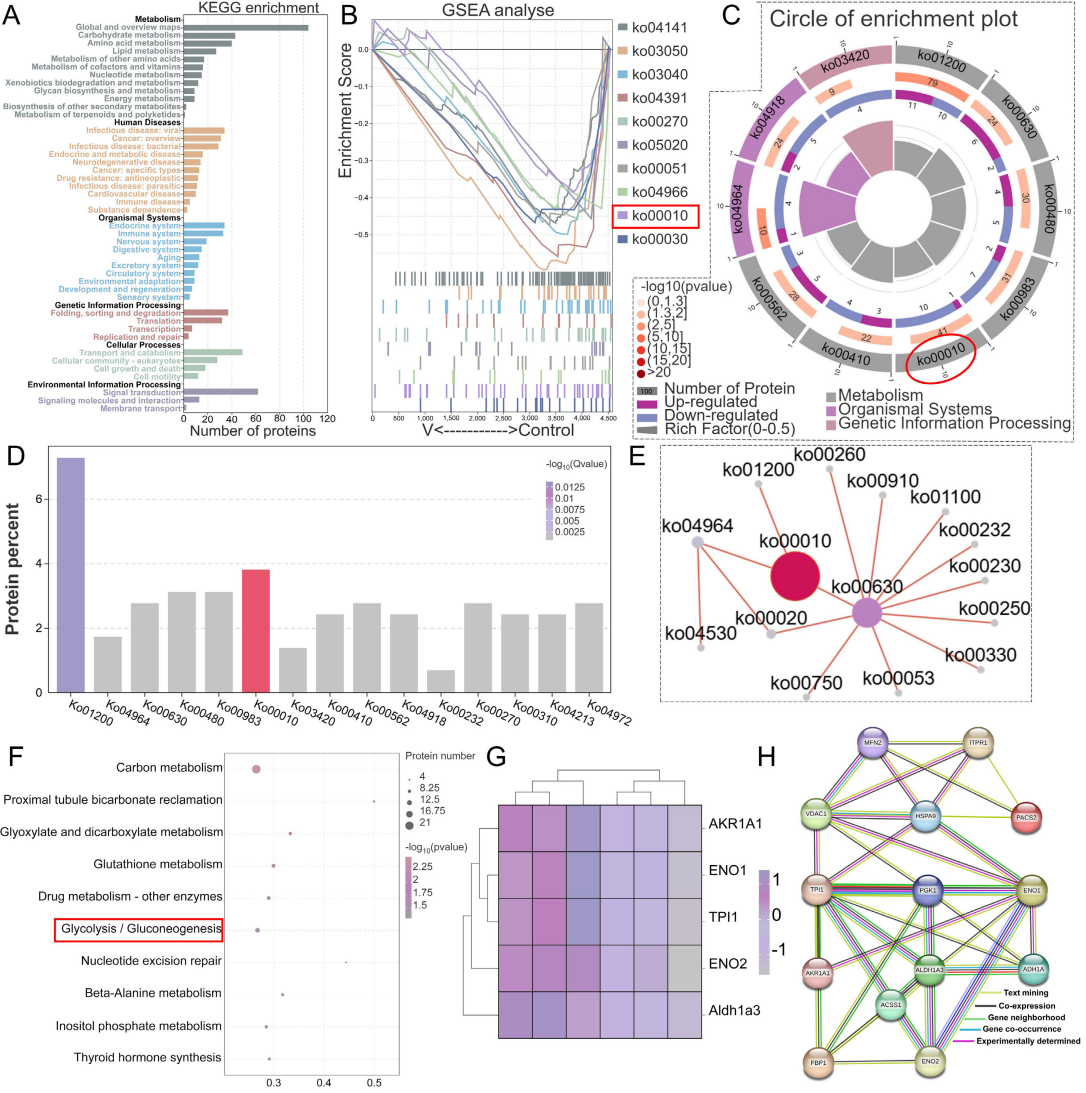
Differences in the proteomic profiles of MAMs induced by excessive V in duck livers. (A) Protein identification and KEGG annotation. (B) GSEA analysis plot of the top 10 KEGG-enriched pathways. (C) Circle of enrichment plot of the top 10 KEGG-enriched pathways. (D) KEGG enriched bar chart. (E) KEGG enrichment pathway network diagram. (F) Functional annotation of biological pathways was performed utilizing the KEGG database for in-depth analysis. (G) Differential proteins heatmap analysis. (H) Exploring PPIs among DEPs involved in glycolysis/gluconeogenesis-related pathway and key proteins of MAMs.

To elucidate the molecular mechanisms underlying the role of differentially expressed proteins (DEPs) in V-evoked mitochondrial-associated membrane (MAMs) dysfunction, we conducted a comprehensive Kyoto Encyclopedia of Genes and Genomes (KEGG) pathway and PPI analyses. In the primary pathway classification based on KEGG, DEPs were distributed across diverse domains, encompassing metabolism, human diseases, organismal systems, cellular processes, genetic information processing, and environmental information processing (Figure 5A). Further in-depth KEGG pathway analysis revealed specific insights into the functional roles of downregulated DEPs in MAMs. These proteins were predominantly involved in various metabolic and regulatory pathways, including glycolysis/gluconeogenesis, carbon metabolism, proximal tubule bicarbonate reclamation, glutathione metabolism, nucleotide excision repair, beta-alanine metabolism, inositol phosphate metabolism, and thyroid hormone synthesis, with interrelated connections existing among these pathways (Figure 5B-F). Notably, glycolysis/gluconeogenesis emerged as a pathway with a close correlation to MAMs function. Given this significant connection, we subsequently performed a PPI proteomic network analysis to further investigate the interactions within this pathway. The results demonstrated that the DEPs involved in glycolysis/gluconeogenesis, namely AKR1A1, ENO1, ENO2, TPI1, Acss1, FBP1, and PGK1, exhibited dense interconnections with key markers of MAMs functionality, including IP3R, Grp75, Mfn2, PACS2, and VDAC1 (Figure 5G-H). These findings suggested that the complex interplay between glycolysis/gluconeogenesis and MAMs function served as a potential regulatory mechanism underlying V-induced hepatotoxicity in ducks.

### 3.6 V induced the glucose homeostasis imbalance in duck livers

**Figure 6:**
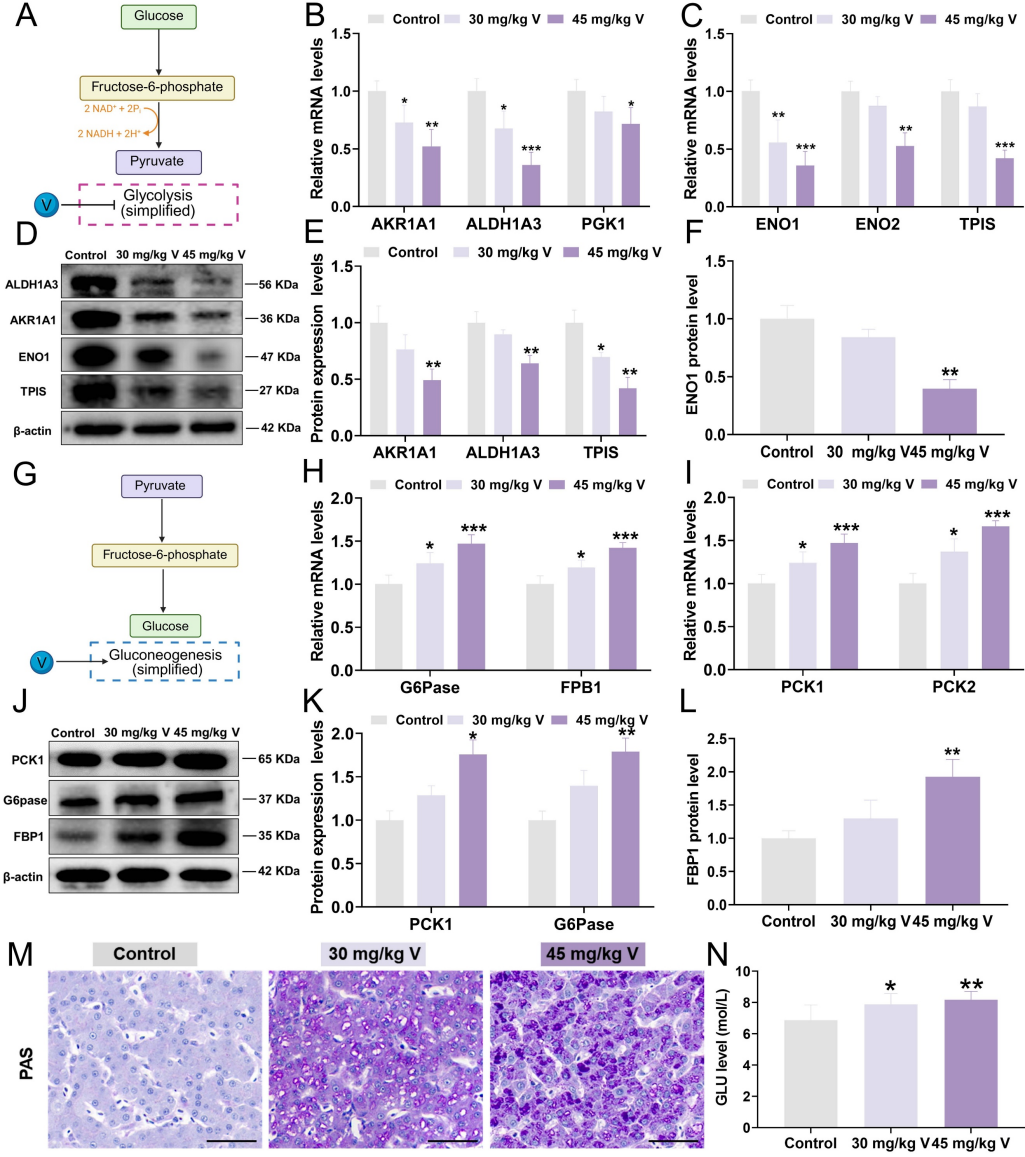
V induced the imbalance of glucose metabolism in duck livers. (A) Glycolysis model diagram. (B) The relative mRNA levels of AKR1A1, ALDH1A3 and PGK1. (C) The relative mRNA levels of ENO1, ENO2 and TPIS. (D) The images of glycolysis associated protein levels (AKR1A1, ALDH1A3, TPIS, ENO1, and β-actin). (E) Gray value analysis of AKR1A1, ALDH1A3 and TPIS proteins. (F) Gray value analysis of ENO1 protein. (G) Gluconeogenesis model diagram. (H)The relative mRNA levels of G6pase and FPB1. (I) The relative mRNA levels of PCK1 and PCK2. (J) The images of gluconeogenesis associated protein levels (PCK1 and G6pase, FPB1 and β-actin). (K) Gray value analysis of PCK1 and G6pase proteins. (L) Gray value analysis of FPB1 protein. (M) Representative images of PAS staining. Scale bar is 50 μm. (N) GLU level.

To gain insights into the interplay between V-induced hepatotoxicity and the MAMs, a panel of DEPs integral to glycolysis/gluconeogenesis was selected for proteomic validation. In brief, glycolysis is the process by which glucose is converted into pyruvate under the catalysis of a series of metabolic enzymes, whereas gluconeogenesis is the reverse process (Figure 6A and 6G). Key molecular markers associated with the glycolysis/gluconeogenesis pathway were subsequently analyzed using RT-qPCR and Western blotting. Notably, the glycolysis-related mRNA levels, including AKR1A1, ALDH1A3, PGK1, ENO1, ENO2, and TPIS, were significantly decreased (*P* < 0.05, *P* < 0.01, or *P* < 0.001) in response to 30 mg/kg and 45 mg/kg V treatments (Figure 6B-C). Consistent with these findings, the protein levels of AKR1A1, ALDH1A3, TPIS, and ENO1 were also markedly down-regulated (*P* < 0.05 or *P* < 0.01) in both V-treated groups compared with the control group (Figure 6D-F). Conversely, the mRNA levels of gluconeogenesis-related mRNA, namely G6Pase, FBP1, PCK1, and PCK2, exhibited a notable elevated (*P* < 0.05 or *P* < 0.001) in both 30 mg/kg and 45 mg/kg V-treated groups (Figure 6H-I). Meanwhile, at the protein level, PCK1, G6Pase, and FBP1 demonstrated a notable up-regulation (*P* < 0.05 or *P* < 0.01) in these V-treated groups compared with the control group (Figure 6J-L). Furthermore, the findings from PAS staining clearly indicated a discernible augmentation in glycogen accumulation in response to escalating doses of V when contrasted with the control group (Figure 6M). Additionally, biochemical assay results further affirmed a significant elevation in blood glucose levels following exposure to V treatment (Figure 6N). The aforementioned results indicated that V induced the glucose homeostasis imbalance by disturbing the glycolysis/gluconeogenesis pathway in duck livers.

### 3.7 V induced ferroptosis via inhibiting NADH/FSP1/CoQ_10_ axis in duck lives

**Figure 7:**
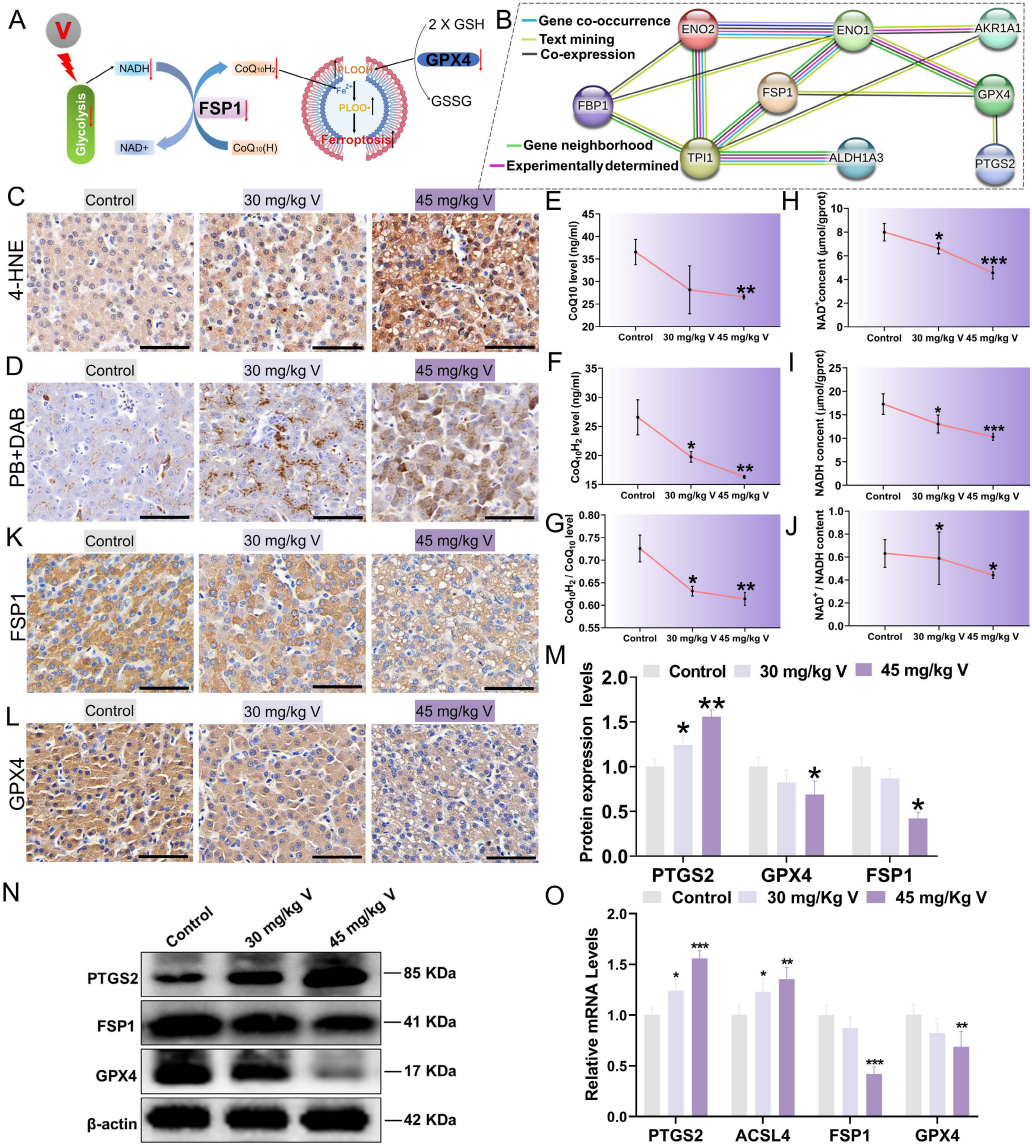
V induced ferroptosis via NADH/FSP1/CoQ_10_ axis in duck lives. (A) The schematic diagram of molecular mechanism of V induced ferroptosis through interference with glycolysis. (B) Exploring PPIs among proteins involved in glycolysis-related pathways and key proteins of ferroptosis. (C) The immunohistochemical staining of 4-HNE. Scale bar is 50 μm. (D) DAB enhanced Prussian blue staining. Scale bar is 50 μm. (E) C_O_Q_10_H_2_ content. (F) C_O_Q_10_ content. (G) The ratio of C_O_Q_10_H_2_ content to C_O_Q_10_ content. (H) NADH content. (I) NAD+ content. (J) The ratio of NADH content to NAD+ content. (K) The immunohistochemical staining of FSP1. Scale bar is 50 μm. (L) The immunohistochemical staining of GPX4. Scale bar is 50 μm. (M) Gray value analysis of PTGS2, FSP1 and GPX4 proteins. (N) The images of ferroptosis associated protein levels (PTGS2, FSP1, GPX4 and β-actin). (O) The mRNA level of GPX4, ACSL4, PTGS2, FSP1.

During the process of inhibiting ferroptosis, FSP1 necessitates the utilization of NADH as an electron donor to facilitate the reduction of C_O_Q_10_(H), subsequently capturing and neutralizing lipid peroxide radicals, thereby safeguarding cells from the peril of ferroptosis (Figure 7A). Additionally, PPI analysis revealed close associations between proteins related to the glycolysis pathway (ALDH1A3, AKR1A1, ENO1, ENO2, and TPIS) and ferroptosis marker proteins (FSP1, GPX4, PTGS2), as depicted in Figure 7B. These findings prompted us to further investigate ferroptosis. Lipid peroxidation stands out as a significant contributor to ferroptosis. Compared with the control group, exposure to 30 mg/kg and 45 mg/kg of V resulted in a marked increase in the areas of 4-HNE-positive and MDA content within liver tissues, accompanied by a notable decrease in GSH content (Figure 7C; Figure S2A, B). Notably, ferroptosis is an iron-dependent process that is often initiated by disruptions in iron metabolism. DAB-enhanced Prussian blue staining results demonstrated a dose-dependent increase in the Fe^3+^ within liver tissues in response to escalating doses of V treatment (Figure 7D). Similarly, the concentrations of both total iron and Fe^2+^ in the livers also increased in a dose-dependent manner with increasing doses of V treatment (Figure S2C-E). To delve deeper into the specific mechanism underlying V-induced ferroptosis, we conducted a comprehensive analysis of the contents of NADH, NAD+, C_O_Q_10_, and C_O_Q_10_H_2_ within liver tissues. Our results indicated that the contents of these substances all exhibited significant decreases, with the decline being more pronounced in the 45 mg/kg V-treated group (Figure 7E-J). Furthermore, immunohistochemical staining of liver sections revealed that V treatment significantly down-regulated the levels of GPX4 and FSP1 in the livers (Figure 7K-L). Consistent with this, as the dosage of V treatment gradually increased, the expression levels of ferroptosis-related markers FSP1 and GPX4 exhibited a significant downward trend, whereas the expression level of PTGS2 showed a notable up-regulation (Figure 7M-O). These results confirmed that V could induce ferroptosis by inhibiting the NADH/FSP1/CoQ_10_ axis in duck livers.

## 4. Discussion

Vanadium (V) is an emerging and potentially hazardous environmental pollutant, whose detrimental effects on biospheric organisms are attracting increasing concern. Organisms are primarily exposed to V through inhalation, ingestion, and direct skin contact. The organism predominantly absorbs V through the digestive tract upon its entry, and it is widely distributed across various tissues and organs, with the liver, as a crucial metabolic and detoxifying organ, being the primary site of its accumulation 31, 32. To comprehensively investigate V-induced hepatotoxicity, this study applied a network toxicology approach. The analysis revealed that V primarily affected biological processes closely associated with organelles, such as hepatocyte biological regulation, stress response, and cellular metabolism. However, previous studies mainly explored V-induced hepatotoxicity mechanisms via individual organelles, potentially missing comprehensive biological effects. As critical functional hubs mediating mitochondria-endoplasmic reticulum interactions, MAMs provide a strategic platform for comprehensively understanding the toxicological mechanisms and biological outcomes of heavy metal exposure. In this study, proteomic sequencing of hepatic MAMs revealed that V could induce MAM-mediated glucose homeostasis imbalance. Importantly, this alteration further inhibited the NADH/FSP1/CoQ_10_ axis, ultimately triggering hepatic ferroptosis in ducks.

Numerous studies have demonstrated that V possessed the ability to directly bind to phospholipids and proteins on hepatocyte membranes, and to interfere with the activities of several crucial enzymes in the liver, thereby impacting the physiological and metabolic functions of the liver and leading to hepatic injury 33, 34. Our results indicated abnormal liver function under V exposure. Additionally, through meticulous ultrastructural observations, we observed an increase in the distance between the ER and mitochondria in the liver under V exposure. Consequently, we guessed that V may induce liver injury by disrupting the structure and function of MAMs. Researches have shown that multiple protein complexes were highly enriched on MAMs, and they acted synergistically to maintain normal intercellular organelle communication and material transport 35-37. Among them, the IP3R-Grp75-VDAC1 complex serves as a direct channel for calcium (Ca^2+^) transport within MAMs, playing a crucial role in maintaining intracellular calcium homeostasis, regulating mitochondrial function, and energy metabolism. Our team previously confirmed that excessive V could up-regulate the levels of factors associated with the IP3R-Grp75-VDAC1 complex in duck renal tubular epithelial cells, leading to MAMs dysfunction, which subsequently elevates mitochondrial Ca^2+^ levels and induces cell death 38. In our study, the fluorescence intensity of the IP3R-Grp75-VDAC1 complex and the expression levels of related factors were increased under V treatment. However, there was a reduction in the number of colocalized spots of the IP3R-Grp75-VDAC1 complex under V treatment. These results confirmed that the functions and connectivity of MAMs were disrupted under V exposure. As we all know, proteomics analysis could reveal changes in the composition, localization, modifications, and interaction patterns of proteins in organisms exposed to heavy metals. For example, Tin Yan Wong et al. 39 employed proteomics analysis to explore in detail the alterations and interaction mechanisms of proteins related to energy metabolism and oxidative stress in the liver under silver nanoparticle (AgNPs) exposure. Building on this foundation, proteomics analysis of intracellular specialized structures holds promise for elucidating the toxic mechanisms of heavy metals from a more microscopic and precise perspective. In light of this, the present study successfully isolated MAMs from duck livers by an improved Percoll gradient method. Subsequently, a comprehensive proteomics analysis was conducted on these MAMs, with the aim of further elucidating the molecular mechanisms underlying V-induced liver injury and delving into the regulatory role of MAMs in this pathological process. After further analyzing DEPs through KEGG enrichment and PPI proteomics network analysis, we selected DEPs within the pathway (glycolysis/gluconeogenesis) closely associated with MAMs for subsequent research.

In the glycolysis pathway, triosephosphate isomerase (TPIS) played a pivotal role by catalyzing the conversion of dihydroxyacetone phosphate (DHAP) to D-glyceraldehyde-3-phosphate (GAP) 40, 41. Subsequently, PGK1 utilized the GAP supplied by TPIS as a substrate to further catalyze its transformation into 3-phosphoglycerate (3-PG), a crucial intermediate that facilitates subsequent glycolytic reactions 42. This process was followed by the conversion of 3-PG to phosphoenolpyruvate (PEP) under the catalysis of enolase 1 (ENO1), ultimately leading to the production of ATP and pyruvate 43. Moreover, AKR1A1 and ALDH1A3, as major multifunctional enzymes within the organism, indirectly supported the normal progression of the glycolytic pathway by reducing various carbonyl compounds within this pathway to alcohols 44-46. In contrast, gluconeogenesis employs an array of gluconeogenic enzymes to catalyze the synthesis of glucose and glycogen from non-carbohydrate precursors. In this process, PCK1 catalyzed oxaloacetic acid (OAA) to produce phosphoenolpyruvate (PEP) 47, which could be further converted into fructose-1,6-diphosphate (FDP) 48, and then fructose-6-phosphate(F6P) under the action of FBP1 49, 50, and finally glucose through the action of G6Pase 50. Heavy metals exhibited a dual mechanism in glucose metabolism, intricately weaving their effects: on the one hand, they exerted an inhibitory influence on the expression levels of metabolic enzymes, such as PGK1, TPIS, and ENO1, thereby subtly impeding the glycolytic process 51-53; on the other hand, they imposed an promoting effect on the expression of enzymes integral to metabolism, encompassing PCK1, G6Pase, and FBP1, thereby effectively enhancing the smooth progression of the gluconeogenic pathway 54-56. These concerted effects ultimately led to increase in blood glucose levels within the organism. In our study, similar alterations were also observed in the expression levels of genes related to glycolysis and gluconeogenesis under V exposure. These results concluded that V induced glucose homeostasis imbalance by inhibiting MAMs-mediated glycolysis and promoting gluconeogenesis, leading to abnormalities in glucose breakdown and synthesis in liver of ducks.

Research showed that the imbalance of glucose homeostasis interfered with the synthesis of various chemicals (NADPH, NADH, and ATP), leading to a series of programmed cell deaths 57. Furthermore, numerous studies have revealed a close link between glucose homeostasis imbalance and metabolite disturbances, as well as a potential association between such disturbances and ferroptosis 58, 59. However, there has been scant investigation into the regulatory mechanisms between glucose homeostasis and the induction of ferroptosis in the research on the toxic mechanisms of heavy metals. In our study, PPI analysis revealed significant interactions between metabolic enzyme proteins in the glycolysis/gluconeogenesis pathway and proteins related to ferroptosis. Based on these observations, we propose that V-induced glucose homeostasis imbalance may further induce ferroptosis in the liver of ducks. Previous studies have shown that glucose homeostasis imbalance led to abnormal accumulation of Fe^2+^, total iron ions and lipid peroxidation products (MDA and 4-HNE) in cells, which precisely constitute the primary factors inducing ferroptosis 60, 61. In addition, the accumulation of lipid peroxidation products caused by the imbalance of glucose homeostasis may further affect the GPX4/GSH system, thus inducing ferroptosis. It is noteworthy that NADH, as one of the major metabolites produced during glycolysis (a primary regulatory pathway for glucose homeostasis), modulates the occurrence of ferroptosis via the NADH/FSP1/C_O_Q_10_ axis. Specifically, FSP1 utilizes NADH as a cofactor to catalyze the reduction of the substrate C_O_Q_10_H (ubiquinone) to C_O_Q_10_H_2_ (ubiquinol). C_O_Q_10_H_2_ is effective in capturing and neutralizing free peroxidation radicals, thereby effectively preventing the occurrence of ferroptosis 62. Therefore, we conjectured that a V-induced imbalance of glucose homeostasis may induce ferroptosis by increasing the accumulation of intracellular Fe^2+^ and lipid peroxidation products, while inhibiting the NADH/FSP1/C_O_Q_10_ axis. Studies have shown that heavy metal exposure had an obvious impact on biological tissues, which is manifested as significantly increased concentrations of MDA, 4HNE and Fe^2+^, decreased GSH content, and down-regulated GPX4 expression in tissues 63, 64. Additionally, prior studies have revealed that heavy metals may indirectly result in a decrease in the NADH/NAD+ ratio by disrupting intracellular metabolic pathways, subsequently inhibiting the functionality of the FSP1/C_O_Q_10_ axis and triggering ferroptosis 65. Our experimental results were similar to those of these studies, indicating that V induced ferroptosis by inhibiting the NADH/FSP1/C_O_Q_10_ axis. Over all, we could confirm that glucose homeostasis imbalance triggered by V exposure further inhibited the NADH/FSP1/C_O_Q_10_ axis, as well as enhanced lipid peroxidation and excessive accumulation of ferrous ions in the liver, which in turn induced ferroptosis in duck livers.

This study integrated network toxicology and targeted MAMs proteomic analysis to reveal a novel mechanism of V-induced hepatotoxicity in ducks. However, the absence of corresponding in vitro experimental validation somewhat limits the reliability of the conclusions. For instance, the failure to establish V exposure model using primary duck hepatocytes made it impossible to directly verify the causal relationship between MAMs structural disruption, glucose metabolism dysfunction, and ferroptosis at the cellular level. Therefore, future studies should employ cell models and molecular biology techniques for targeted intervention experiments to further corroborate the proposed mechanistic pathway.

## 5. Conclusion

In the present study, we employed quantitative proteomics to delve into the alterations in the protein expression profile of MAMs in duck livers under excessive V exposure. Our research findings indicated that V could induce glucose homeostasis imbalance mediated by MAMs, thereby inhibiting the NADH/FSP1/C_O_Q_10_ axis and ultimately leading to ferroptosis in duck livers. This discovery underscores the pivotal role of MAMs in liver damage induced by V exposure and offers novel insights into the understanding of V-induced hepatotoxicity.

## Supplementary Material

Supplementary materials and methods, figure and table.

## Figures and Tables

**Table 1:** Primers.

Gene	Forward Primer	Reverse Primer
β-actin	ATGTCGCCCTGGATTTCG	ATGTCGCCCTGGATTTCG
IP3R	AGACACCTTTGCACTAACACC	TTTGCCGTTCTCTATTGGGTT
VDAC1	TGTTCCACCTGCTTATGCTG	AGCCTGTCTTAACTTTAGCG
Grp75	CGGTCACTAACCCACACAACACC	AGTCAGGTCAACGCCCGTCT
Mfn2	CTGGCATTGATGTAACCAC	CAAAGAAAATTCGATCCCCT
PACS2	GATTGCCACCACTCCGACCA	TGCAAATCAACCTGCTGATGCC
AKR1A1	CGTCCCTCATTCTCGTGTGTGT	GGGCAGGTGACTTGCTGTAT
ALDH1A3	AGCAATAACCCCGTGGAACTT	TTGATGGGCACACTCCACTG
PGK1	CTGGAACTGCTGGAGGGTAA	CCCTGCCCAGTTTCGGTTTA
ENO1	CGCTACATGGGGAAAGGTGT	AAACTGTCAGCACCACAGGG
ENO2	ACACAGCATCTCCCCTTGAG	TAGACTTCAGCCCCAATGCG
TPIS	TGGAAGATGAACGGCGACAA	CTGTGAAAGCACCCTTTGGC
G6pase1	GGTGGTGAAGGGTTAAGGCA	ACCATCACGTAGTACACGCC
FBP1	TCATCACCATGACCCGCTTC	CCACAAGATTACGCCCTGGT
PCK1	TCGACACACTGATGAACGGG	TTGCGGGGTGGATCGTTTTA
PCK2	CACATGCTGATTTTGGGGGTG	GGTCCATGATGGGACACTGG
FSP1	GACTACAGATGAGCAGACAAGTG	TGTACACATAAGCTCCTGCCC
GPX4	ATCCTTGCTGCTCAAACCCA	GTCATTCGTGCTGGGTGAGA
PTGS2	ATCCTTGCTGCTCAAACCCA	ACGTGAAGAATTCCGGTGTTG
ACSL4	GCGTGTGCTCTCCTCTTCTA	AAGTTCACAAAAATTCGGTGCT

Sequences of target genes primer. All sequences are shown 5′→ 3′.
