# Supplementary material for: Dual-pathway mechanism of vanadium-induced hepatotoxicity in ducks: Synergistic crosstalk between glucose homeostasis disruption and NADH/FSP1/COQ10 axis-driven ferroptosis
Source: Int J Biol Sci. 2026 Jan 1;22(1):43–59. doi: 10.7150/ijbs.123482 (PMC12681742; doi:10.7150/ijbs.123482)
Supplement: Supplementary file 1 — Supplementary materials and methods, figure and table. [file ijbsv22p0043s1.pdf]

Supplementary Information 1

1 MAMs extraction method

Firstly, crude mitochondria were extracted from approximately 2 g of liver tissue. Immediately after dissection, the tissues were washed three to four times with pre-cooled $IB_{liver-1}$ and $IB_{liver-3}$ solutions. The liver was then minced in ice-cold $IB_{liver-1}$ solution and homogenized using a glass pestle on ice. For initial separation, two centrifugation steps were carried out at 740 g for 5 min each at 4 °C. The supernatant was collected and subjected to further centrifugation at 9000 g for 10 min for purification. The resulting pellet was gently resuspended in ice-cold $IB_{liver-2}$ solution, and centrifugation at 10,000 g was repeated twice, allowing the suspension to stand for 10 min after each resuspension. Finally, the pellet was resuspended in a small volume of ice-cold mitochondrial resuspension buffer (MRB). The suspension containing crude mitochondria was carefully layered onto approximately 8 mL of a Percoll–MRB gradient medium and centrifuged at 95,000 g for 40 min using a Beckman Coulter Optima XPN ultracentrifuge (Beckman, USA) equipped with an SW41 rotor. After centrifugation, the pure mitochondrial fraction was located at the bottom of the tube, while the middle layer contained the mitochondrial-associated membranes (MAMs). The MAMs fraction was gently diluted in MRB and centrifuged at 6300 g for 10 min at 4 °C. The resulting supernatant was then centrifuged at 100,000 g for 70 min at the same temperature to pellet the MAMs. Finally, the purified MAMs pellet was gently resuspended in MRB for subsequent use.

2 MAMs extraction reagent and components

MAMs-related reagents were formulated according to the method of Wieckowski et al (Wieckowski, Giorgi, Lebiedzinska, Duszynski & Pinton, 2009). All the prepared solutions were ready-made for use.

Table S1 MAMs extraction reagent and components

Reagent	Content
Startingbuffer (SB)	225 mM-mannitol, 75 mM-sucrose, 30 mM Tris-HCl (pH 7.4)
$IB_{liver-1}$	225 mM-mannitol, 75 mM-sucrose, 0.5% BSA, 0.5-mM EGTA, 30-mM Tris-HCl (pH7.4)
$IB_{liver-2}$	225 mM-mannitol, 75 mM-sucrose, 0.5% BSA, 30-mM Tris-HCl (pH 7.4)
$IB_{liver-3}$	225-mM mannitol, 75-mM sucrose and 30-mM Tris-HCl pH 7.4
MRB	250 mM-mannitol, 5 mM-HEPES (pH 7.4), 0.5-mM EGTA
Percoll medium	225 mM-mannitol, 25-mM-HEPES (pH 7.4), 1-mM EGTA, 30% Percoll

References:

Wieckowski, M. R., Giorgi, C., Lebiedzinska, M., Duszynski, J., & Pinton, P. (2009). Isolation of mitochondria-associated membranes and mitochondria from animal tissues and cells. *Nature Protocols*, *4*(11), 1582-1590.

4 Figure S1

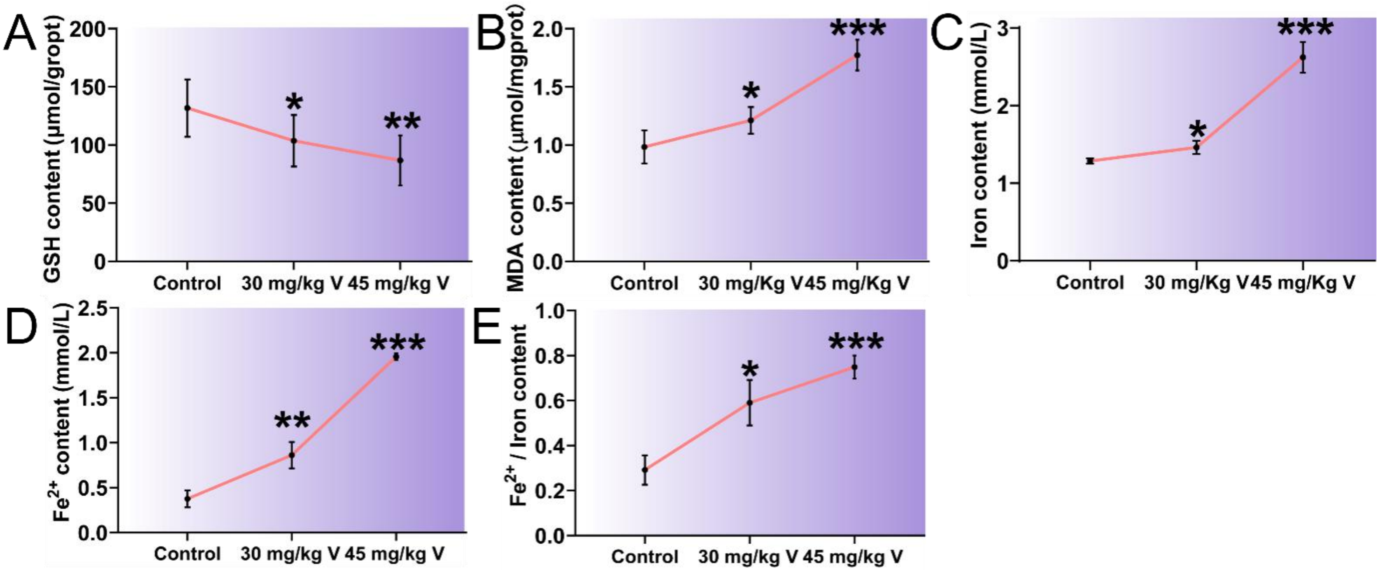

Figure S1 (A) GSH content. (B) MDA content. (C) Iron content. (D) Fe^{2+} content. (E) The ratio of Fe^{2+} content to Iron content content.
